# Supplementary material for: Transcriptomics and Immunological Analyses Reveal a Pro-Angiogenic and Anti-Inflammatory Phenotype for Decidual Endothelial Cells
Source: Int J Mol Sci. 2019 Mar 31;20(7):1604. doi: 10.3390/ijms20071604 (PMC6479455; doi:10.3390/ijms20071604)
Supplement: Supplementary file 1 [file ijms-20-01604-s001.pdf]

Supplementary Materials

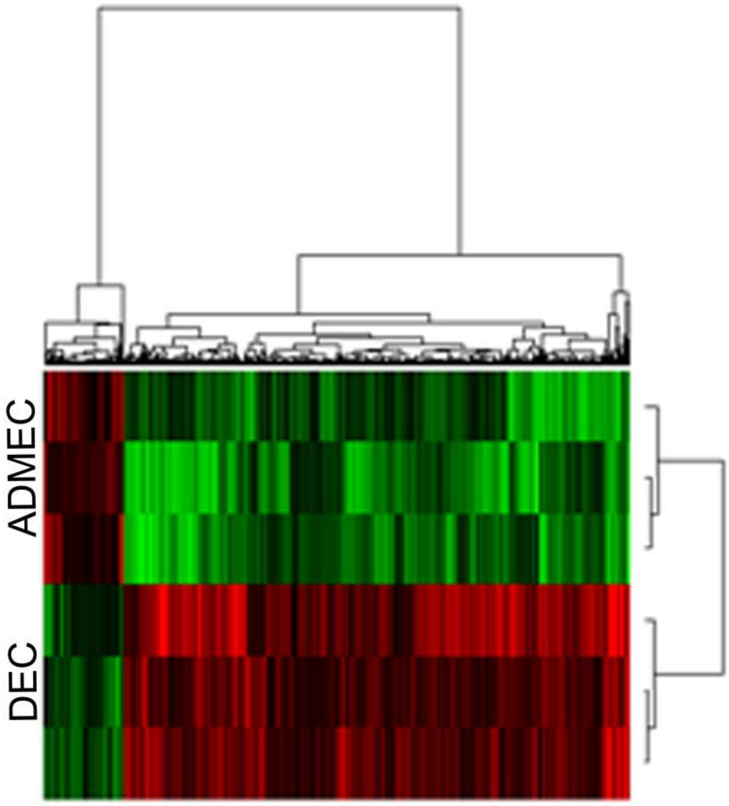

Gene Symbol	Accession number	Regulation	Fold Change
HGF	NM_004712	up	2,137
VEGF-A	NM_003376	up	2,66
IGFBP3	NM_001013398	up	11,601
ICAM2	NM_000873	up	5,742
ICAM3	NM_002162	up	3,76

Supplemental Figure S1. Overview of gene expression patterns of three populations of DECs and ADMECs. Heatmap of the microarray cluster of 1909 genes. Genes up-regulated and down-regulated in DECs were selected based on fold-change (≥1 or < 1).

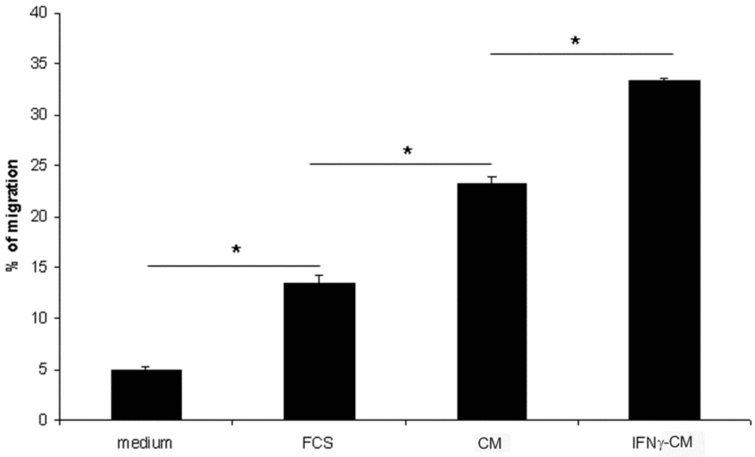

Supplemental Figure S2. Effect of the conditioned media of DECs treated with IFN-γ on the transendothelial migration of LM. LM were added to the upper compartment of the TW inserts and allowed to migrate through DECs. FCS were used as a positive control, while medium without serum was used as negative control. The data are expressed as percentage of LM migrated through the endothelium and represent the mean ± SD of triplicate samples from five separate experiments. * $p < 0.05$.

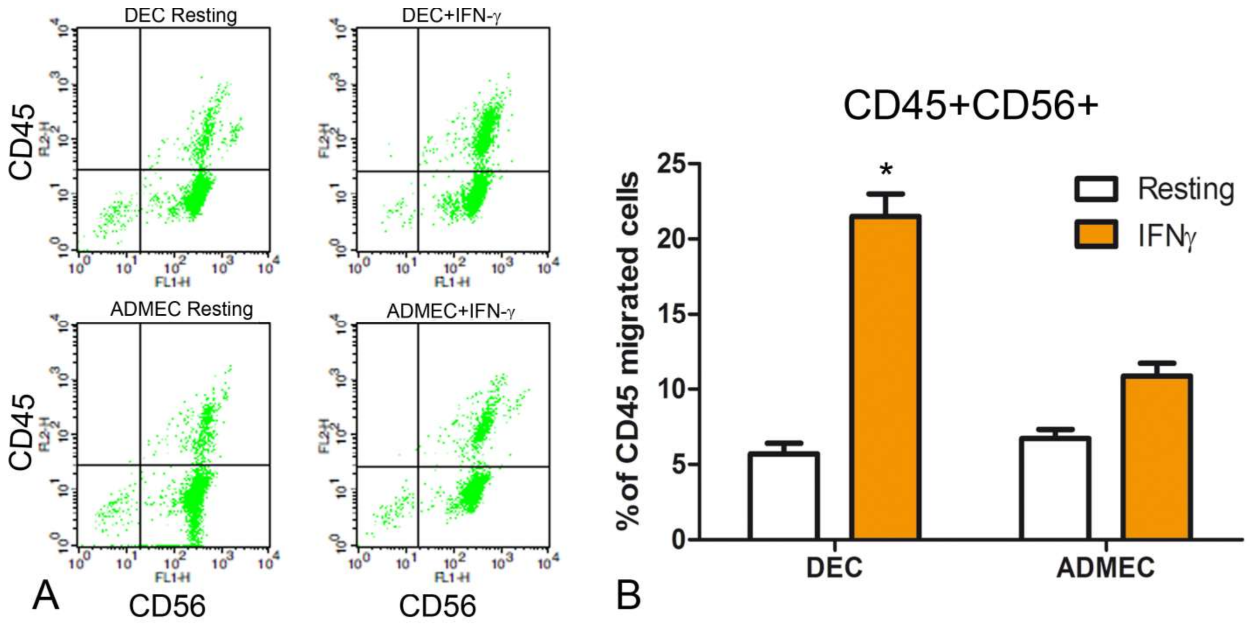

Supplemental Figure S3. Transendothelial migration of PBDM across untreated and IFNγ-treated DEC and ADMEC. (A) Representative dot plots for flow cytometry of trans-endothelial migrated LM, stained for CD45 and CD56. (D) Quantitation of the percent of total migrated LM cells, positive for both CD45 and CD56 by flow cytometry. The data represent the mean ± SD of duplicate samples from three separate experiments * $p < 0.05$.

Supplemental Table S1. Primer used for qPCR analysis.

Gene	Primers	Sequence 5′>3′	Annealing Temperature (°C)	Amplicon Size (bp)	Gene Bank Accession Number
18S	Forward	atccctgaaaagttccagca	60	154	NM_022551
	Reverse	ccctcttggtgaggtcaatg			
HGF	Forward	AACGACAAGAACCCACACGTC	62	182	NM_004712
	Reverse	GGCCTGGATCAGGTACAGGA			
VEGF-A	Forward	GCAGAATCATCACGAAGTGG	59	212	NM_003376
	Reverse	GCATGGTGATGTTGGACTCC			
IGFBP3	Forward	CCCTCCATTCAAAGATAATC	62	298	NM_001013398
	Reverse	TCCACACACCAGCAGAAG			
ICAM-2	Forward	ATCTCCCATGACACGGTCCT	60	162	NM_002162
	Reverse	CACCCTGCACTCAATGGTGAA			
ICAM-3	Forward	GTGTTTGAAGGAAGGCTCC	60	113	NM_002162
	Reverse	TGTATTTGCCTCGTGAGCTG			
